# Zinc Supplementation Enhances the Hematopoietic Activity of Erythropoiesis-Stimulating Agents but Not Hypoxia-Inducible Factor–Prolyl Hydroxylase Inhibitors

**DOI:** 10.3390/nu16040520

**Published:** 2024-02-13

**Authors:** Akira Takahashi

**Affiliations:** Dialysis Center, Tesseikai Neurosurgical Hospital, Shijonawate 575-8511, Japan; kinnereth@gaia.eonet.ne.jp; Tel.: +81-72-877-6639; Fax: +81-72-877-6692

**Keywords:** erythropoiesis-stimulating agent, erythropoiesis-stimulating agent responsiveness index, hypoxia-inducible factor–prolyl hydroxylase inhibitors, hemodialysis, renal anemia, zinc deficiency

## Abstract

Since zinc is involved in many aspects of the hematopoietic process, zinc supplementation can reduce erythropoiesis-stimulating agents (ESAs) in patients undergoing hemodialysis. However, it remains unclear whether hypoxia-inducible factor–prolyl hydroxylase inhibitors (HIF-PHIs) have similar reduction effects. HIF-PHI stabilizes HIF, which promotes hematopoiesis, although HIF-1α levels are downregulated by zinc. This study aimed to investigate the effect of zinc supplementation on the hematopoietic effect of HIF-PHI in patients undergoing hemodialysis. Thirty patients undergoing maintenance hemodialysis who underwent periods of treatment with roxadustat or darbepoetin alfa during the past 3 years were retrospectively observed. Participants who underwent periods with and without zinc supplementation were selected, with nine treated with darbepoetin alfa and nine treated with roxadustat. Similarly to the ESA responsiveness index (ERI), the hematopoietic effect of zinc supplementation was determined by the HIF-PHI responsiveness index (HRI), which was calculated by dividing the HIF-PHI dose (mg/week) by the patient’s dry weight (kg) and hemoglobin level (g/L). Zinc supplementation significantly increased ERI (*p* < 0.05), but no significant change was observed (*p* = 0.931) in HRI. Although zinc supplementation did not significantly affect HRI, adequate zinc supplementation is required to alleviate concerns such as vascular calcification and increased serum copper during the use of HIF-PHI.

## 1. Introduction

Zinc supplementation has been observed to reduce the dosage of erythropoiesis-stimulating agents (ESAs) in patients on dialysis [1,2]. Since 2019, hypoxia-inducible factor–prolyl hydroxylase inhibitors (HIF-PHIs), such as roxadustat, have been introduced as oral treatments for renal anemia. However, it remains unclear whether zinc supplementation influences the hematopoietic effects of HIF-PHIs.

Most patients undergoing dialysis require zinc supplementation due to zinc deficiency [3]. Although HIF-PHIs can now be used to treat renal anemia, there are potential concerns, which include malignancy and retinopathy due to the activation of the vascular endothelial growth factor (VEGF) and cyst growth in polycystic kidneys. Moreover, side effects such as thrombosis and hypothyroidism have been reported, and recommendations for proper use have been issued because of the reported side effects [4]. Furthermore, the benefits of combining zinc supplementation with HIF-PHI use have been reported to address these concerns and the side effects associated with HIF-PHI use. The use of HIF-PHIs stabilizes hypoxia-inducible factor 1α (HIF-1α) activity, raising concerns about the activation of VEGF and vascular calcification [5,6]. However, vascular calcification is reportedly suppressed by zinc [7]. Roxadustat usage has been associated with hypothyroidism [8,9], which is potentially induced by its T3-like allosteric effects [10] and can be partially alleviated by zinc supplementation [11]. Zinc supplementation, initiated to restore immune function also improved thyroid function (as evidenced by a decrease in thyroid-stimulating hormone levels) and led to increased T3 and T4 concentrations [12], suggesting a regulatory effect of zinc on thyroid hormones. Therefore, concurrent zinc supplementation is recommended when using roxadustat.

Since HIF-PHI administration increases serum copper concentrations, zinc supplementation during HIF-PHI administration can stabilize copper concentrations, emphasizing the need for adequate zinc supplementation during HIF-PHI use [13]. However, patients prescribed high doses of zinc can develop iatrogenic copper deficiency [14]; thus, the proper balance of zinc and copper concentrations is important. However, there have been many reports of concerns about a decrease in serum copper concentration due to zinc supplementation [15,16]. In patients undergoing hemodialysis, if serum zinc concentration exceeds 18.4 µmol/L (120 μg/dL), the serum copper concentration will fall below the standard value [13,15]. Conversely, it has been reported that an increase in the Cu/Zn ratio may further exacerbate inflammation [17], and higher Cu/Zn ratios appeared to be associated with higher Type 2 diabetes risk [18].

As mentioned above, an ESA-reducing effect has been observed with zinc supplementation, but it is unclear whether a similar effect occurs with the use of HIF-PHIs. Both HIF-1α [19] and HIF-2α [20,21] are involved in hematopoiesis. While zinc reportedly downregulates HIF-1α levels [22], there is no report describing whether zinc downregulates HIF-2α levels, which precede erythropoietin transcription. If zinc not only downregulates HIF-1α levels but also inhibits the involvement of both HIF-1α and HIF-2α in hematopoiesis, the hematopoietic effect of HIF-PHI by zinc supplementation may remain unchanged or weakened. However, if zinc enhances the hematopoietic effect of HIF-PHI, this suggests that zinc does not inhibit HIF-2α. The aim of this study is to investigate the impact of zinc supplementation on the hematopoietic effects of HIF-PHI in patients undergoing hemodialysis in a setting where zinc is suspected to have an inhibitory effect on HIF.

## 2. Methods

### 2.1. Participants

This retrospective observation study was performed on 56 outpatients who were undergoing maintenance hemodialysis in the morning, and included 30 patients (14 females and 16 males; mean age of 78.0 ± 15.6 years) treated with both periods of HIF-PHI (roxadustat) and ESA (darbepoetin alfa) from October 2020 to September 2023. Patients with malignancies, ongoing or prior treatment for diabetic retinopathy, or cases of polycystic kidney disease were avoided due to the potential concerns of HIF-PHI treatment, and patients with iron deficiencies, which are associated with thromboembolic events, were also excluded [4]. Patents who had liver dysfunction and hemorrhagic lesions were excluded from the study. Patients who did not require treatment for renal anemia with ESA (darbepoetin alfa) or HIF-PHI were not included in this study.

Among the included patients, nine participants were selected for a comparison of the effects of zinc supplementation during periods when roxadustat was used over 3 years. These selected participants experienced on and off zinc supplementation periods during the time they were using roxadustat.

**Figure 1:**
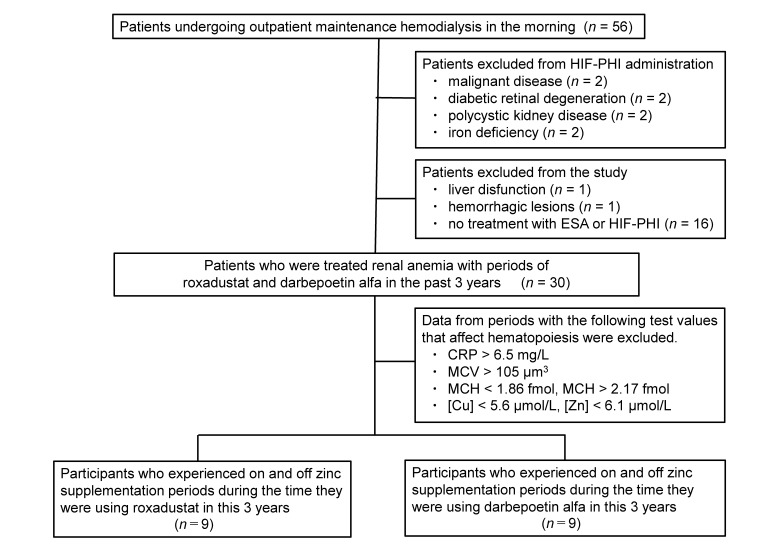
Study design. ESA, erythropoiesis-stimulating agent (darbepoetin alfa); HIF-PHI, hypoxia-inducible factor–prolyl hydroxylase inhibitor (roxadustat); CRP, C-reactive protein; MCV, mean corpuscular volume; MCH, mean corpuscular hemoglobin; [Cu], serum copper concentration; [Zn], serum zinc concentration.

Similarly, during the same 3-year period, nine participants who had used darbepoetin alfa were identified. These patients experienced on and off zinc supplementation periods during the time they were using darbepoetin alfa (Figure 1).

All participants received standard dietary advice for patients undergoing hemodialysis, but no further advice was provided regarding their diet before the commencement of hemodialysis.

### 2.2. Data Collection

Blood samples were collected at the beginning of the week before hemodialysis. The initiation or modification of medication was carried out from the end of the hemodialysis day of that blood collection week. Therefore, roxadustat administrations and zinc supplements that had been ongoing before each blood collection were considered to be involved in the data collected.

In our cohort of patients undergoing hemodialysis, serum zinc and copper concentrations were measured every 3 months as part of routine examinations starting in 2018. Hematological data related to anemia measured at the same time as these serum zinc and copper concentrations were extracted from these measurements. C-reactive protein (CRP), white blood cell count, red blood cell count, hemoglobin, hematocrit, mean corpuscle volume (MCV), mean corpuscular hemoglobin (MCH), platelet, red cell distribution width standard deviation (RDW-SD), red cell distribution width coefficient of variation (RDW-CV), reticulocytes, serum iron, total iron-binding capacity (TIBC), transferrin saturation (TSAT), and serum ferritin, were measured via routine clinical chemistry procedures using commercial kits. Serum zinc concentrations were measured using a direct colorimetric assay, based on the nitro-PAPS method, using a JCA-BM6050 BioMajesty (JEOL Ltd., Tokyo, Japan) and ESPA ZnII (Nipro Co., Ltd., Osaka, Japan) reagent. Serum copper concentrations were measured using a direct colorimetric assay, based on the 3,5-DiBr-PAESA method, and the JCA-BM6050 BioMajesty and Quick Auto Neo Cu (Shino-Test, Tokyo, Japan) were used as the reagents. Blood counts were measured twice monthly, and serum levels of iron-associated parameters, zinc, and copper were measured once every three months.

Data from periods with the following test values that affect hematopoiesis were excluded. Elevated C-reactive protein (CRP > 6.5 mg/L) was excluded because hematopoietic function is impaired during CRP elevation [23]. If MCV > 100 µm^3^, macrocytic anemia is suspected [24]. In this study, an MCV > 105 µm^3^ was excluded. Data during MCH abnormality, indicating iron deficiency when below 1.86 fmol [25] and suggesting folate or vitamin B12 deficiency when above 2.17 fmol [26], were excluded. Because hypocupremia affects hemoglobin levels, serum copper concentrations below 5.6 µmol/L (35.5 µg/dL), which is half the lower normal limit, were excluded. Zinc supplementation was given to patients with low serum zinc concentrations, but those with serum zinc concentrations of 6.1 µmol/L (40 µg/dL), half of the lower normal limit, were also excluded.

For the prevention of iron deficiency anemia, it is recommended to initiate iron supplementation when serum ferritin is lower than 100 µg/L and TSAT is lower than 20% [27]. In this study, data with TSAT lower than 18% were excluded, as these values were deemed to impact the assessment of effectiveness.

The ESA Responsiveness Index (ERI) indicates a hematopoietic effect during the use of darbepoetin alfa. ERI was defined as the weekly ESA dose divided by clinical dry weight and blood hemoglobin level, as previously described [1,2], using the following equation: ERI = [weekly ESA dose (μg/week)]/[dry weight (kg)]/[hemoglobin (g/L)]. Similarly to ERI, the hematopoietic effect of zinc supplementation in HIF-PHI users was determined by the HIF-PHI Responsiveness Index (HRI), which is calculated by dividing the HIF-PHI dose (mg/week) by dry weight (kg) and blood hemoglobin (g/L), employing the following equation: HRI = [weekly HIF-PHI dose (mg/week)]/[dry weight (kg)]/[hemoglobin (g/L)].

### 2.3. Statistical Analysis

Data are presented as the mean ± standard deviation for continuous variables and percentages with counts for categorical variables. Microsoft 365 Excel (Microsoft Corporation, Redmond, WA, USA) was utilized for data analysis. Baseline characteristics of the two groups were tested to determine whether they were normally distributed using the Shapiro–Wilk test [28], followed by a comparison using a two-sample equal variance Student’s *t*-test. Categorical data were compared using the chi-square test. The relationship between continuous variables was assessed using Pearson’s product–moment correlation coefficient calculated with the CORREL function, and *p* values were obtained using the TDIST function. Statistical significance was set at *p* < 0.05.

### 2.4. Treatment Protocol

ESA consisted of darbepoetin alfa (Kyowa Kirin Co., Ltd., Tokyo, Japan; injection at doses of 10, 20, 30, and 40 μg) which were administered intravenously after a weekend hemodialysis session, while HIF-PHI included roxadustat (Astellas Pharma Inc., Tokyo, Japan; tablets at doses of 20, 50, and 100 mg) which was administered three times weekly during hemodialysis sessions to ensure adherence. Administered doses of iron and roxadustat were adjusted according to the Recommendations of the Asian Pacific Society of Nephrology (APSN) on the appropriate use of HIF-PH inhibitors [4] and the guidelines for renal anemia in chronic kidney disease [29]. During the administration of darbepoetin alfa and roxadustat, iron supplementation was provided to prevent iron deficiency when the serum ferritin concentration was less than 100 μg/L or transferrin saturation was less than 20%.

The proper balance of zinc and copper concentrations is important, as patients prescribed high doses of zinc can develop iatrogenic copper deficiency. Since 2018, our institution has measured serum zinc and copper concentrations every 3 months as part of routine blood tests for patients undergoing hemodialysis. Based on this experience, we have adopted the following zinc supplementation protocol. Participants with zinc deficiency (serum zinc concentration < 9.2 μmol/L) were initiated on zinc supplementation with zinc acetate hydrate (C_4_H_6_O_4_Zn.2H_2_O) or polaprezinc (C_9_H_12_N_4_O_3_Zn), and the dose was adjusted based on the severity of the zinc deficiency. Administered doses included zinc acetate hydrate at 25 mg/day (zinc content 25 mg), polaprezinc at 150 mg/day (zinc content 34 mg), zinc acetate hydrate at 50 mg/day (zinc content 50 mg), and zinc acetate hydrate at 100 mg/day (zinc content 100 mg). If the serum zinc concentration exceeded 15.3 µmol/L (100 μg/dL), the dose was reduced to zinc acetate hydrate at 25 mg/day. If the serum zinc concentration exceeded 18.4 µmol/L (120 μg/dL), zinc supplementation was discontinued because the serum copper concentration would fall below the normal range [15]. The target serum zinc concentration was set as 12.2–18.4 µmol/L (80–120 μg/dL). Normal ranges for serum copper concentrations were 11.2–20.8 µmol/L (71–132 µg/dL) [30].

## 3. Results

### 3.1. Characteristics of Participants

Participants who experienced periods both with and without zinc supplementation included nine participants during the darbepoetin alfa period and nine participants during the roxadustat period. The background characteristics of each participant are presented in Table 1.

### 3.2. Data for Patients Using Darbepoetin Alfa or Roxadustat without Zinc Supplements

For periods when participants in the darbepoetin alfa and roxadustat groups were not receiving zinc supplementation, data for each variable (except for serum copper and serum zinc concentrations) showed no significant differences, as indicated in Table 2 and Table 3. Zinc supplementation amounts for the darbepoetin alfa and roxadustat groups were 36.5 ± 8.9 mg/day and 35.9 ± 10.2 mg/day, respectively, with no significant difference between groups (*p* = 0.87).

### 3.3. Data for Participants Using Darbepoetin Alfa with and without Zinc Supplements

Data for the darbepoetin alfa group are shown in Table 2. ERI during the non-zinc supplementation period was 0.0034 ± 0.0023 μg/week/kg/g/L, while it was 0.0018 ± 0.0017 μg/week/kg/g/L during the zinc supplementation period. Similarly to previous reports [1,2], a significant decrease in ERI due to zinc supplementation was observed (*p* < 0.05).

ESA usage associated with zinc supplementation decreased significantly from 18.1 ± 10.9 to 9.9 ± 8.1 μg/week (*p* < 0.05), with an average ESA reduction rate of 45.4%/week.

### 3.4. Data for Participants Using Roxadustat with and without Zinc Supplements

Data for the roxadustat group are shown in Table 3. HRI during the non-zinc supplementation period was 0.025 ± 0.017 mg/week/kg/g/L, and during the zinc supplementation period it was 0.025 ± 0.010 mg/week/kg/g/L, with no significant changes in HRI due to zinc supplementation (*p* = 0.957).

**Figure 2:**
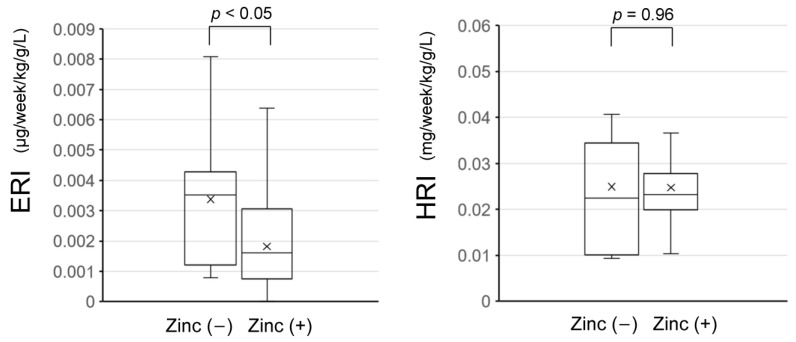
Comparison of ERI and HRI with and without zinc supplementation. ERI obtained from data for the darbepoetin alfa group are shown in the left box-and-whisker plot. A significant decrease in ERI due to zinc supplementation was observed (*p* < 0.05). HRI obtained from data for the roxadustat group are shown in the right box-and-whisker plot. No significant change in HRI due to zinc supplementation was observed (*p* = 0.96). The X in the box represents the mean. The median divides the box into the interquartile range. The box represents 50% of the data set, distributed between the first and third quartiles. The lines (whiskers) running vertically outside the box indicate the range of outliers outside the upper and lower quartiles, the lowest and highest data points in the data set. ERI, erythropoiesis-stimulating agent responsiveness index; HRI, hypoxia-inducible factor–prolyl hydroxylase inhibitor responsiveness index.

Graphs of ERI and HRI are shown in Figure 2.

### 3.5. Relationship between Zinc Supplementation Dose and HRI

The evaluation of the relationship between the zinc supplementation dose and the HRI of participants showed no correlation between these two variables (*r* = −0.037, *p* = 0.800).

### 3.6. Relationship between Serum Zinc Concentrations and HRI

The evaluation of the relationship between serum zinc concentrations and the HRI of the participants showed no correlation between these two variables (*r* = −0.258, *p* = 0.070).

### 3.7. Serum Copper and Zinc Concentrations in Participants Using Darbepoetin Alfa or Roxadustat

Serum copper was significantly higher in the roxadustat group compared with the darbepoetin alfa group (*p* < 0.05).

Serum zinc significantly increased during the zinc supplementation periods in both darbepoetin alfa and roxadustat groups (*p* < 0.05).

## 4. Discussion

It was previously reported that zinc supplementation can lead to a decrease in ERI [1,2]. In this study, whether a similar effect could be observed for HIF-PHIs, such as roxadustat, was investigated. However, zinc supplementation did not result in a significant change in HRI for roxadustat.

Serum copper concentrations in patients participating in this study were significantly higher in the roxadustat group compared with the darbepoetin alfa group. The possible reasons for this include the following: As previously reported [13,31], the use of HIF-PHI increases serum copper concentrations. Moreover, in this study, when arranged chronologically by patients, serum copper concentrations significantly increased from 12.2 ± 1.4 μmol/L to 17.7 ± 1.9 μmol/L after switching from darbepoetin alfa to roxadustat (*p* < 0.05). Since the serum copper concentration was measured every 3 months, it would naturally have increased if the patient had changed to roxadustat before the measurement.

When the serum copper concentrations with respect to roxadustat use were arranged in chronological order for each patient, the serum copper concentration decreased in all participants after the initiation of zinc supplementation, although the change was not significant. Therefore, it is necessary to start zinc supplementation with caution and to check serum zinc and copper concentrations at least every 3 months to adjust the amount of zinc supplementation [15].

**Figure 3:**
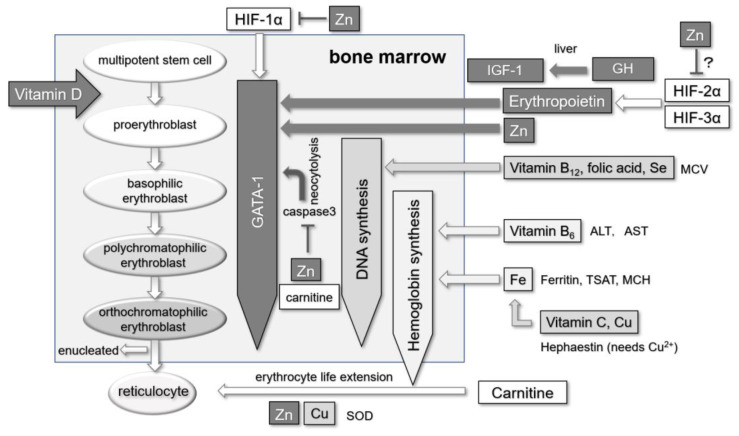
Overview of the involvement of zinc, copper, HIF, and various factors in the process of erythropoiesis [15]. In the initial phase of erythropoiesis (upper right, dark gray boxes/arrows), hematopoiesis is initiated through the collaborative action of Epo and IGF-1 [33]. IGF-1 is generated through the influence of GH, with both GH and IGF-1 also exhibiting a connection to zinc. In the initial phase of erythropoiesis (upper right, dark gray boxes/arrows), hematopoiesis is initiated through the collaborative action of Epo and IGF-1 [34]. As multipotent stem cells transition to proerythroblasts (upper left), Epo binds to the Epo receptor, leading to the release of GATA-1, an essential erythroid differentiation factor (center, dark gray boxes/arrows) [35]. In the context of HIF’s role in hematopoiesis, HIF-2α predominantly regulates Epo production [43] with HIF-3α also playing a role [45], while HIF-1α induces the expression of GATA-1 [44]. Although there are reports indicating that zinc downregulates HIF-1α levels [22], there is currently no information on whether zinc similarly affects HIF-2α or HIF-3α levels (upper right, ? mark). Proerythroblasts undergo transformations into basophilic erythroblasts, polychromatophilic erythroblasts, and orthochromatophilic erythroblasts (left). GATA-1 features a zinc finger in its structure [36]. Upon the cessation of or reduction in Epo, caspase 3 is activated, leading to the degradation of GATA-1 and neocytolysis. However, the actions of carnitine and zinc inhibit caspase 3, preventing apoptosis (center, dark gray boxes/arrows) [37,38,39]. Vitamin D serves as an adjunct in the early phases of erythroid differentiation (upper left) [40]. The vitamin D receptor also incorporates a zinc finger as a structural component [41]. Upon the release of GATA-1 and the commencement of erythroid differentiation, DNA synthesis initiates, necessitating vitamin B12 and folic acid (center right, lighter gray arrows/boxes) [47,48]. Concurrently, hemoglobin synthesis commences, with vitamin B6 playing a crucial role in the initial stages of this synthesis (bottom right, lightest gray arrows/boxes) [46]. Hephaestin requires copper for its function [54]. Following the formation of reticulocytes, copper/zinc superoxide dismutase acts as a scavenger [42], while carnitine extends the lifespan of erythrocytes (bottom, white boxes/arrows) [39]. This figure has been modified and reprinted with permission [15]. ALT, alanine aminotransferase; AST, aspartate aminotransferase; Cu, copper; DNA, deoxyribonucleic acid; Epo, erythropoietin; Fe, iron; GH, growth hormone; HIF, hypoxia-inducible factor; IGF-1, insulin-like growth factor-1; MCH, mean corpuscular hemoglobin; MCV, mean corpuscular volume; SOD, superoxide dismutase; TSAT, transferrin saturation; Zn, zinc.

An overview of the involvement of zinc, copper, HIF, and various factors in the process of erythropoiesis is shown in Figure 3. Zinc plays a crucial role in various stages of the hematopoietic process [15,32]. In the pre-stage, where erythropoietin acts, the growth hormone (GH) acts on insulin-like growth factor 1 (IGF-1), initiating hematopoiesis with erythropoietin and IGF-1 [33]. Both GH and IGF-1 require zinc [34]. After erythropoietin binds to the erythropoietin receptor, the erythroid transcription factor GATA-1 (a zinc finger protein transcription factor that regulates various genes involved in RBC synthesis [35]) is released, which contains a zinc finger in its structure [36]. When erythropoietin is stopped or the dose is reduced, caspase 3 is released and it mediates the degradation of GATA-1 to cause neocytolysis, but zinc and carnitine inhibit caspase 3 and prevent apoptosis [37,38,39]. Vitamin D functions as an adjuvant during the early stages of RBC differentiation [40], and the vitamin D receptor also has a zinc finger in its structure [41]. Once reticulocytes are formed, copper/zinc superoxide dismutase acts as a scavenger [42], extending the lifespan of RBCs (Figure 3).

Given the significant role of zinc in the hematopoietic process, zinc supplementation improved ERI when conventional ESA medications were used in the treatment of renal anemia. Concerning HIF’s role in hematopoiesis, erythropoietin production is mainly predominantly regulated by HIF-2α [43], while HIF-1α induces the expression of GATA-1 [44], a pivotal factor in erythroid differentiation. The involvement of HIF-3α in erythropoietin production has also been demonstrated [45]. Since the use of HIF-PHI causes the stabilization of HIF, HIF-2α promotes erythropoietin production, and HIF-1α promotes GATA-1 production, which in turn promotes hematopoiesis. Meanwhile, zinc has been reported to downregulate HIF-1α levels [22]. Because zinc supplementation did not alter the hematopoietic effects of HIF-PHI, this suggests that zinc may inhibit not only HIF-1α but also HIF-2α, which are both involved in hematopoiesis.

In this study, the improvement in hematopoietic function due to zinc administration was not observed when using HIF-PHI. However, this does not imply that zinc supplementation is unnecessary during HIF-PHI use. The factors contributing to ESA hyporesponsiveness include not only iron deficiency but also zinc deficiency.

Other deficiencies in elements essential for erythropoiesis include insufficiencies in vitamin B6, vitamin B12, folic acid, vitamin C, copper, carnitine, and others [29]. In clinical practice, the suspicion of ESA deficiency arises when the RBC count falls below 3.0 10^12^/L, while iron deficiency is considered if MCH is less than 1.86 fmol (30 pg) [25]. Low values of alanine transaminase (ALT) or aspartate transaminase (AST) suggest a vitamin B6 deficiency that is crucial for heme synthesis [46]. Elevated MCV indicates a potential deficiency in vitamin B12 or folic acid deficiency [47,48]. Selenium deficiency, which also manifests as macrocytic anemia, is suspected in the absence of abnormal values for vitamin B12 or folic acid, alongside low protein levels and decreased thyroid function [49].

Carnitine and zinc deficiencies are common in dialysis patients due to inadequate intake, impaired absorption, and excessive loss [15,50,51]. Approximately 80% of carnitine is eliminated during a single hemodialysis session. As a result, carnitine levels are assessed every 6 months to ensure the maintenance of normal circulating concentration of free carnitine (36–74 µmol/L). The supplementation of carnitine is adjusted to achieve a predialysis-free carnitine concentration of 180 μmol/L or higher [52,53]. Zinc levels are adjusted to ensure a serum concentration between 12.2 and 18.4 µmol/L, with the simultaneous monitoring of serum copper concentration [13].

When iron is pumped from ferroportin, copper is required for hephaestin [54]. In iron-deficient patients, intravenous iron supplementation, as opposed to oral supplementation, results in non-physiological iron supplementation and further elevates hepcidin. Increased hepcidin can worsen impaired iron utilization and escalate oxidative stress and infection risk, leading to ESA hyporesponsiveness [55,56]. The use of HIF-PHI, which inhibits hepcidin, has been shown to improve ESA hyporesponsiveness [57].

HIF-1α induces ceruloplasmin [58] and transferrin [59], and it is known that zinc ions are essential when iron undergoes a change from divalent to trivalent states by ceruloplasmin and is subsequently transferred to transferrin [60].

In 2016, it was reported that HIF-1α strongly promotes the calcification of vascular smooth muscle cells induced by inorganic phosphate [6]. In 2020, Nagy et al. reported that this calcification of vascular smooth muscle cells is suppressed by zinc [7]. When a HIF-PHI (roxadustat) was added to the growth medium of human aortic smooth muscle cells, the number of calcified areas stained red with alizarin red increased. When zinc was added, the red calcified parts disappeared. Although this was an in vitro study, the results show that zinc supplementation is increasingly necessary in this era of using HIF-PHI for renal anemia.

Additionally, during the use of HIF-PHI, there is an increase in serum copper concentrations. Transporters involved in iron metabolism, such as ferroportin, duodenal cytochrome B (DCYTB), and divalent metal transporter 1 (DMT1) are induced by HIF-2α [57,61,62]. These transporters not only transport Fe^2+^ but also divalent cations such as Zn^2+^, Mn^2+^, Cu^2+^, and Co^2+^. Furthermore, copper is absorbed by being reduced from being divalent to monovalent in the duodenum. HIF-2α increases the expression of copper transporter 1 (CTR1) [63,64], and ATPase (Atp7a) [65]. Therefore, there was a concern that serum copper concentration would increase when using HIF-PHI. Excessive increases in serum zinc and copper concentrations are also implicated in dementia [66]. However, this concern about serum copper concentrations is stabilized by the reductive effect of appropriate zinc supplementation [13].

Regarding serum copper concentrations, previous reports suggest that hypocupremia causes Alzheimer’s disease [67] and people with high copper concentrations have a lower risk of Alzheimer’s disease [68]. However, the excessive intake of copper can have detrimental effects on the brain [69], with some reports showing that patients with Alzheimer’s disease had significantly higher serum copper concentrations than healthy controls [70]. Serum copper concentrations reportedly increase when HIF-PHIs are used [31]. Therefore, there is concern that long-term HIF-PHI use may cause persistent hypercupremia, which may lead to the future development of dementia. However, with proper zinc supplementation, excessive increases in serum copper concentrations can be prevented [16,26]. Notably, there are reports that zinc inhibits amyloid-β aggregation [71], and controversial reports show that zinc promotes amyloid-β aggregation [72,73]. Therefore, zinc supplementation to adjust serum copper concentration must be carried out appropriately to prevent both hypocupremia and hyperzincemia.

While zinc supplementation may not improve hematopoietic effects during the use of HIF-PHIs, sufficient zinc supplementation is recommended to address concerns related to vascular calcification and the increase in serum copper concentration associated with HIF-PHI use. As there was no correlation between zinc supplementation and HRI, it is advisable to set the zinc dosage in consideration of both serum copper and zinc concentrations to prevent excess levels during the use of HIF-PHI.

This study had some limitations. First, since 2018, serum zinc and copper concentrations have been measured as routine tests, and patients with hypozincemia have been actively given zinc supplementation. Therefore, by the time roxadustat started being used, many patients had already started using HIF-PHI with zinc supplementation. Although there were 30 users of roxadustat, only 9 ultimately participated, as the final analysis was performed on patients who experienced both periods of zinc supplementation and no zinc supplementation, resulting in a decrease in the number of data points.

Furthermore, recommendations regarding the appropriate use of HIF-PHIs suggest choosing HIF-PHI rather than ESA when the cause of ESA hyporesponsiveness is unknown [4]. However, in the cohort of this study, countermeasures against ESA hyporesponsiveness as described above were already implemented. Therefore, in reality, zinc supplementation during the use of HIF-PHI not only did not change the hematopoietic function caused by HIF-PHI, but it may have worsened it.

## 5. Conclusions

Since zinc plays an important role in the process of hematopoiesis, zinc supplementation improves ERI during darbepoetin alfa administration. The use of roxadustat stabilizes HIF-1α, HIF-2α, and HIF-3α. Erythropoietin, which is involved in hematopoiesis, is regulated by HIF-2α and HIF-3α, and GATA-1 (erythroid transcription factor) is induced by HIF-1α. It has been reported that at least HIF-1α is inhibited by zinc. Therefore, zinc supplementation did not significantly impact HRI during roxadustat administration. However, adequate zinc supplementation is essential when administering roxadustat to prevent concerns and side effects such as vascular calcification, hypothyroidism, and excessive serum copper increase.

## Figures and Tables

**Table 1:** Baseline characteristics of participants.

	ESA	HIF-PHI	*p* Value
Number of participants	9	9	
Male sex, *n* (%)	5 (55.5)	4 (44.4)	0.637
Age, mean ± SD (years)	74.5 ± 11.4	77.6 ± 11.9	0.608
Duration of dialysis, mean ± SD (years)	7.8 ± 4.3	5.2 ± 2.2	0.187
Etiology, *n* (%)			0.776
Diabetic nephropathy	4 (44.4)	4 (44.4)	
Nephrosclerosis	3 (33.3)	4 (44.4)	
Chronic glomerulonephritis	1 (11.1)	1 (11.1)	
Other	1 (11.1)	0 (0.0)	

ESA: participants who had periods both with and without zinc supplementation during treatment with darbepoetin alfa; HIF-PHI: participants who had both with and without zinc supplementation periods during treatment with roxadustat.

**Table 2:** Data obtained in the group of participants using darbepoetin alfa.

	Normal Range	Unit	Zinc (−)			Zinc (+)			*p*-Value
Zinc		mg/day	0.0	±	0.0	36.5	±	8.9	
ERI		μg/week/kg/g/L	0.0034	±	0.0023	0.0018	±	0.0017	0.046
ESA		μg	18.1	±	10.9	9.9	±	8.1	0.028
ESA/week		μg/week	18.1	±	10.9	9.9	±	8.1	0.028
Dry weight		kg	54.7	±	10.0	54.1	±	8.2	0.857
CRP	0.0–1.4	mg/L	0.83	±	0.59	0.66	±	0.55	0.441
White blood cell	3.3–8.6	10^9^/L	6.26	±	1.47	5.73	±	1.44	0.327
Red blood cell	4.35–5.55	10^12^/L	3.36	±	0.33	3.41	±	0.27	0.636
Hemoglobin	137–168	g/L	106.2	±	9.9	106.0	±	6.3	0.939
Hematocrit	0.41–0.50		0.326	±	0.031	0.326	±	0.022	0.978
MCV	83.6–98.2	µm^3^	97.4	±	4.4	95.9	±	3.7	0.336
MCH	1.71–2.06	fmol	1.97	±	0.07	1.94	±	0.07	0.255
Platelet	158–348	10^9^/L	185.5	±	52.7	194.1	±	52.7	0.659
RDW-SD	39–46	fL	48.4	±	4.7	45.8	±	2.9	0.159
RDW-CV	0.116–0.146		0.144	±	0.018	0.138	±	0.008	0.351
Reticulocytes	0.50–2.00	%	1.80	±	1.00	1.38	±	0.82	0.220
Serum iron	7.2–33.7	μmol/L	12.8	±	5.2	13.3	±	2.8	0.522
TIBC	43.9–72.0	μmol/L	42.2	±	6.4	45.3	±	7.6	0.254
TSAT		%	33.21	±	8.62	29.45	±	6.21	0.196
Serum ferritin	13–277	μg/L	160.1	±	192.2	92.5	±	65.0	0.222
Serum copper	11.2–20.8	μmol/L	13.25	±	2.43	12.4	±	2.8	0.872
Serum zinc	12.2–19.9	μmol/L	9.48	±	1.59	12.7	±	3.0	0.00081

Zinc (+): data obtained during the period with zinc supplementation; Zinc (−): data obtained during the period without zinc supplementation; Zinc: dose of zinc supplementation; ERI, ESA responsiveness index; ESA, erythropoiesis-stimulating agent (darbepoetin alfa); CRP, C-reactive protein; MCV, mean corpuscular volume; MCH, mean corpuscular hemoglobin; RDW-SD, red cell distribution width standard deviation; RDW-CV, red cell distribution width coefficient of variation; TIBC, total iron-binding capacity; TSAT, transferrin saturation.

**Table 3:** Data obtained in the group of participants using roxadustat.

	Normal Range	Unit	Zinc (−)			Zinc (+)			*p*-Value
Zinc		mg/day	0.0	±	0.0	35.9	±	10.2	
HRI		mg/week/kg/g/L	0.025	±	0.017	0.025	±	0.010	0.957
HIF-PHI		mg	49.2	±	26.4	50.3	±	16.9	0.874
HIF-PHI/week		mg/week	147.7	±	79.3	151.0	±	50.7	0.874
Dry weight		kg	57.4	±	8.4	58.2	±	11.6	0.820
CRP	0.0–1.4	mg/L	1.09	±	1.42	1.01	±	0.90	0.830
White blood cell	3.3–8.6	10^9^/L	6.93	±	1.48	6.83	±	1.32	0.655
Red blood cell	4.35–5.55	10^12^/L	3.22	±	0.21	3.29	±	0.21	0.317
Hemoglobin	137–168	g/L	101.1	±	6.6	105.5	±	6.5	0.050
Hematocrit	0.41–0.50		0.31	±	0.02	0.32	±	0.02	0.231
MCV	83.6–98.2	µm^3^	96.3	±	3.3	96.3	±	3.3	0.760
MCH	1.71–2.06	fmol	1.95	±	0.08	1.99	±	0.08	0.203
Platelet	158–348	10^9^/L	191.0	±	53.5	188.2	±	44.3	0.862
RDW-SD	39–46	fL	48.5	±	7.1	50.9	±	8.5	0.436
RDW-CV	0.116–0.146		0.14	±	0.02	0.15	±	0.03	0.576
Reticulocytes	0.50–2.00	%	2.01	±	1.19	1.73	±	0.69	0.359
Serum iron	7.2–33.7	μmol/L	11.9	±	2.9	13.6	±	5.0	0.297
TIBC	43.9–72.0	μmol/L	45.8	±	6.6	46.9	±	8.4	0.697
TSAT		%	27.0	±	7.7	28.5	±	7.5	0.571
Serum ferritin	13–277	μg/L	148.0	±	120.3	120.3	±	105.5	0.212
Serum copper	11.2–20.8	μmol/L	17.8	±	3.7	17.8	±	3.3	0.849
Serum zinc	12.2–19.9	μmol/L	8.0	±	1.9	11.8	±	3.7	0.0016

Zinc (+): data obtained during the period with zinc supplementation; Zinc (−): data obtained during the period without zinc supplementation; Zinc: dose of zinc supplementation; HRI: HIF-PHI responsiveness index; HIF-PHI: hypoxia-inducible factor–prolyl hydroxylase inhibitor (roxadustat); CRP, C-reactive protein; MCV, mean corpuscular volume; MCH, mean corpuscular hemoglobin; RDW-SD, red cell distribution width standard deviation; RDW-CV, red cell distribution width coefficient of variation; TIBC, total iron-binding capacity; TSAT, transferrin saturation.

## Data Availability

The data that support the findings of this study will be made available by the corresponding author upon reasonable request.

## Abbreviations

CRP: C-reactive protein; DMT1, divalent metal transporter 1; ERI, ESA responsiveness index; ESA, erythropoiesis-stimulating agent; HIF-PHI, hypoxia-inducible factor–prolyl hydroxylase inhibitor; HRI, HIF-PHI responsiveness index; MCH, mean corpuscular hemoglobin; MCV, mean corpuscular volume; RDW-CV, red cell distribution width-coefficient of variation; RDW-SD, red cell distribution width-standard deviation; TIBC, total iron-binding capacity; TSAT, transferrin saturation.
